# Functional differentiation and scalable production of renal proximal tubular epithelial cells from human pluripotent stem cells in a dynamic culture system

**DOI:** 10.1111/cpr.13190

**Published:** 2022-01-31

**Authors:** Thao Thi Thanh Ngo, Bella Rossbach, Isabelle Sébastien, Julia C. Neubauer, Andreas Kurtz, Krithika Hariharan

**Affiliations:** ^1^ BIH Center for Regenerative Therapies Charité Universitätsmedizin Berlin Berlin Germany; ^2^ Project Centre for Stem Cell Process Engineering Fraunhofer Institute for Biomedical Engineering (IBMT) Würzburg Germany; ^3^ Fraunhofer Institute for Biomedical Engineering (IBMT) Berlin Germany

**Keywords:** alginate, bioreactor, functional tubular epithelial cells, kidney differentiation, Pluripotent stem cells

## Abstract

**Objective:**

To provide a standardized protocol for large‐scale production of proximal tubular epithelial cells (PTEC) generated from human pluripotent stem cells (hPSC).

**Methods:**

The hPSC were expanded and differentiated into PTEC on matrix‐coated alginate beads in an automated levitating fluidic platform bioLevitator. Differentiation efficacy was evaluated by immunofluorescence staining and flow cytometry, ultrastructure visualized by electron microscopy. Active reabsorption by PTEC was investigated by glucose, albumin, organic anions and cations uptake assays. Finally, the response to cisplatin‐treatment was assessed to check the potential use of PTEC to model drug‐induced nephrotoxicity.

**Results:**

hPSC expansion and PTEC differentiation could be performed directly on matrix‐coated alginate beads in suspension bioreactors. Renal precursors arose 4 days post hPSC differentiation and PTEC after 8 days with 80% efficiency, with a 10‐fold expansion from hPSC in 24 days. PTEC on beads, exhibited microvilli and clear apico‐basal localization of markers. Functionality of PTECs was confirmed by uptake of glucose, albumin, organic anions and cations and expression of KIM‐1 after Cisplatin treatment.

**Conclusion:**

We demonstrate the efficient expansion of hPSC, controlled differentiation to renal progenitors and further specification to polarized tubular epithelial cells. This is the first report employing biolevitation and matrix‐coated beads in a completely defined medium for the scalable and potentially automatable production of functional human PTEC.

## Graphical abstract

A robust, reproducible protocol that uses biolevitation and hPSC on matrix‐coated alginate beads for scalable and potentially automatable production of functional human PTEC in a completely defined serum‐free and xeno‐free medium with minimal factors. In this article, we provide evidence of these hPSC‐derived renal progenitors maturing to give rise to polarized functional proximal tubule epithelial cells capable of endocytosis, as well as injury response under distinct cultivation conditions. We therefore demonstrate a platform that offers an excellent starting point for a flexible and scalable production with minimum human intervention and reduced variability, well suited to cater the needs of future cell therapies.

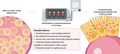

## 1 INTRODUCTION

The kidney has a crucial role in blood clearance, homeostasis maintenance, and waste product elimination. Through renal arteries, the blood enters the kidney where it is passively filtered in the glomeruli, followed by selective reabsorption of between 70% and 100% of the substances in the pre‐urine, including water, amino acids, electrolytes, and glucose by proximal tubular epithelial cells (PTEC) via numerous transport systems.^1^ PTEC are cuboidal, mononuclear cells with apical‐basal polarization and densely microvilli covered brush borders on the apical side, a typical morphological feature distinguishing PTEC and distal tubular cells.^2^ PTEC are also responsible for detoxification and secretion of exogenous compounds such as drugs and xenobiotics into the urine.

Due to high susceptibility to toxic and waste compounds, PTEC are extremely vulnerable, and their injury may result in renal failure or total destruction. There is a high demand of PTEC for tissue modeling, large‐scale drug‐induced nephrotoxicity screening, and potentially for regenerative therapies. Immortalized and primary PTEC cultivated as 3‐dimensional microtissue were reported to dedifferentiated within 10 days.^3^ Moreover, although exhibiting a variety of functional transporters, primary PTEC are variable depending on donors, and partially dedifferentiate *in vitro* while immortalized PTEC lines show functional changes related to the immortalization procedures.^4^ To improve PTEC‐models, several differentiation protocols of human pluripotent stem cells (hPSC) into PTEC have been developed.^4^, ^5^, ^6^ The use of human induced pluripotent stem cells (hiPSC) provides an unlimited source of cells that are donor specific and can be genetically modified to present specific kidney disease backgrounds. However, general limitations of these hPSC‐derived PTEC are often their immature transporter properties, limited polarization and short lifespan. A lack of technologies for efficient, robust and automatable mass production of high quality PTEC limits their applicability.

Many biomaterials have been developed for expansion, embedding, and differentiation of stem cells.^7^ Alginate hydrogel, for example, offers a variety of advantages such as low cost, environmental friendliness, high biocompatibility, low cytotoxicity, easy purification, functionalization, and adjustable gelation.^8^ Spherical alginate beads after coating with extracellular matrix allow cultivation of cells and expansion of surfaces by bead supplementation.^9^ In addition, the beads are easily applied to fluidic culture systems of diverse designs including stirring, rotating, and agitation bioreactors. These fluidic systems can be adapted to mimic the fluidic environment of the proximal tubule epithelia and may increase polarization, barrier, and transport functions of PTEC.^10^ In 2015, Elanzew and colleagues first reported long‐term expansion of hPSC as undifferentiated aggregates with low inoculation density using a BioLevitator^TM^, now CERO from OLS.^11^ In 2017, expansion of human stem cells on Matrigel‐coated alginate beads using this system was reported.^9^

We used a biolevitation‐based approach allowing scalable cell culture to expand and differentiate hPSC into human PTEC cultivated on Matrigel‐coated alginate beads. The biolevitation together with the floating cell‐covered beads models a fluidic environment. This system supported the efficient expansion of hPSC and their immediate differentiation to renal progenitors in a single system. This is the first report of using biolevitation of cell‐coated alginate beads for scalable and potentially automated production of functional human PTEC.

## 2 MATERIALS AND METHOD

### 2.1 Cell culture and maintenance in static culture

The hiPSC lines WISCi004‐A (referred to as IMR90‐4‐iPS, derived from female lung fibroblast) from passages 35 to 65, BCRTi005^12^ derived from urinary cells at passage 25 to 35, and WAe001‐A derived from male blastocyst were cultured on 6‐well plates (Falcon) coated with Geltrex (Thermo Fisher Scientific) in serum‐free, defined Essential 8 (E8) medium (STEMCELL Technologies). Cells were maintained in a humidified 5% CO_2_ atmosphere at 37°C. 0.5mM ethylenediaminetetraacetic acid (EDTA Gibco) in calcium/magnesium free phosphate‐buffered saline (PBS) was used to passage hPSC as colonies.

### 2.2 Bioreactor platform

BioLevitator^T^ ^M^, now CERO from Omni Life Science GmbH & Co was designed as a sealed miniaturized incubator, where parameters for cell culture including CO_2_ level, temperature, speed of tube rotation, and thus, fluidic stress can be adjusted. It can manage separately four 50ml vessels called Levitubes^TM^, now CEROtubes^TM^ with a maximum working capacity of 50ml each.

### 2.3 Preparation, seeding and expansion of hPSC on Matrigel‐coated alginate beads

Matrigel‐coated alginate beads or Matrigel‐coated beads were supplied by Fraunhofer Institute for Biomedical Engineering (IBMT), Project Centre for Stem Cell Process Engineering, Würzburg, Germany. Growth factor‐reduced Matrigel was used to cover alginate beads. Matrigel‐coated beads were stored at 4°C until use. Before hPSC seeding, Matrigel‐coated beads were rinsed twice with E8 medium. Around 40cm^2^ Matrigel‐coated beads were used for each 50 ml CEROtubes^TM^. Confluent hPSC were harvested using 0.5mM EDTA and reseeded on Matrigel‐coated beads at a density of 2.6 × 10^6^ cells in a final volume of 4ml E8 medium for each tube. On the next day, E8 was filled up to 10ml and changed every day. 4 days after seeding, 90% of Matrigel‐coated beads were covered by cells. Observation of beads and cells on beads was performed with phase contrast microscopy (Nikon Eclipse Ts2).

### 2.4 PTEC differentiation and expansion

**FIGURE 1:**
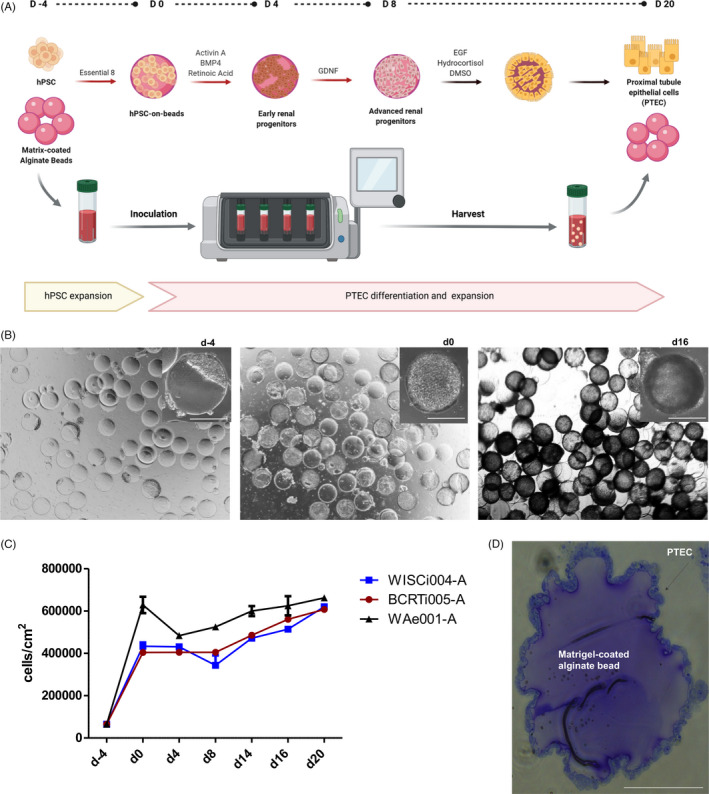
Expansion and differentiation of hPSC into PTEC on Matrigel‐coated alginate beads by biolevitation. (A) Schematic illustration of hPSC expansion and PTEC differentiation on Matrigel‐coated alginate beads by biolevitation. The protocol includes two definite steps: (i) Expansion of hPSC in Essential 8 medium for 4 days from d4 to d0; (ii) Differentiation and Expansion of PTEC from d0 to d20. This step includes (1) differentiation of hPSC into renal progenitors for 8 days and (2) generation, expansion and maintenance of PTEC; (B) Phase contrast pictures of cells on Matrigel‐coated alginate beads on d4, d0, d16. One representative bead for each time point was taken with scale bar = 500µm; (C) Growth rate of cells on 40 cm^2^ of Matrigel‐coated alginate beads from seeding (d‐4) until d20 for the hiPSC lines (WISCi004‐A, BCRTi005‐A) and the embryonic stem cell (ESC) line WAe001‐A, respectively (n = 3); (D) Toluidine blue stained histological section of a Matrigel‐coated alginate bead at d16 with a PTEC monolayer on the bead surface. Scale bar = 100µm

Confluent hPSC on Matrigel‐coated beads were differentiated into renal progenitors following the protocol developed by Hariharan et al.^6^ Specifically, hPSC were induced into intermediate mesoderm during the first 4 days in STEMdiff™ APEL2™ Medium (Stemcell Technologies) with 5% Protein free hybridoma medium (PFHMII) in presence of 10ng/ml Activin A (Peprotech), 1µM Retinoic Acid (Sigma‐Aldrich), and 30ng/ml recombinant human bone morphogenetic protein 4 (BMP4) (Peprotech). This step was followed by further 4 days in the same basal medium supplemented with 150ng/ml Glial derived neurotrophic factor (GDNF) (Peprotech) (Figure 1(A)).

To induce differentiation and expansion of PTEC, an optimized low glucose, serum‐free medium, called PTEC medium was replaced on day 8 (d8). Basal medium composition of PTEC medium included a mixture of low glucose Gibco ^TM^ DMEM (11054) and Ham's F‐12K (21127022, Thermo Fisher) in a 1:1 ratio, then this mixture was mixed to Defined Keratinocyte‐serum‐free medium (KSFM) (10785–012**, Gibco) in a 1:1 ratio. This basal medium was supplemented with Insulin‐Transferrin‐Selenium (ITS) (Gibco), 10ng/ml Epidermal growth factor (EGF), 1µM Hydrocortisone and 0.5% Dimethyl sulfoxide (DMSO). Cells were maintained in this medium until day 20 (d20) (Figure 1(A)). PTEC medium was changed every other day. The differentiation was monitored by marker expression analysis on day 4 (d4) and day 8 (d8) for renal progenitors, and on days 10 (d10), 12 (d12), 14 (d14), 16 (d16), 20 (d20) for PTEC.

In static culture of hPSCs, the differentiation protocol for renal progenitors and PTEC was performed with identical media and timelines with the exception that Geltrex was used for coating instead of Matrigel. For matrix comparison, renal progenitor cells were harvested on day 8 using Accutase cell dissociation reagent (Gibco), re‐plated on Laminin521 (LN521) (BioLamina) and differentiated into PTEC.

### 2.5 Immunofluorescence staining

Cultured cells on 96‐well plates were washed with PBS, fixed with Cytofix (BD Biosciences) for 10 minutes at room temperature (RT). Afterward, cells were washed twice with PBS, blocked with 10% secondary antibody host serum for 30 minutes at RT and further incubated with primary antibodies overnight at 4°C. Primary antibodies were diluted in BD Perm/Wash™ with dilution factor 1:100 or following the manufacturer's instructions. After washing twice with BD Perm/Wash™, labeled secondary antibodies were applied to the cells in 1:1000 dilution for 1h at RT in the dark. Finally, the cells were stained with fluorescent dye 4′, 6‐diamidino‐2‐phenylindole (DAPI; Sigma‐Aldrich, D‐8417) to label nuclei. Negative controls omitted either primary or both primary and secondary antibodies. The primary antibodies using in this study were Aquaporin 1 (AQP1) (Proteintech), Sodium‐Potassium ATPase (Na⁺/K⁺‐ATPase) (Abcam), Sodium‐Glucose cotransporter‐2 (SGLT2) (Abcam), Megalin/LRP2 (Abcam), E‐Cadherin (BD Biosciences), and Kidney injury molecular‐1 (KIM‐1) (Thermo Fisher). Secondary antibodies were Donkey anti‐Mouse IgG (H + L) Secondary Antibody, Alexa Fluor 488; Donkey anti‐Mouse IgG (H + L) Secondary Antibody, Alexa Fluor 647; Donkey anti‐Rabbit IgG (H + L) Secondary Antibody, Alexa Fluor 488; Donkey anti‐Rabbit IgG (H + L) Secondary Antibody, Alexa Fluor 647 (all from Thermo Fisher). Biotinylated Lotus Tetragonolobus Lectin (LTL) (Vector Laboratories), Texas Red^TM^‐X Phalloidin (Thermo Fisher) were used to detect Fucose on microvilli and actin filaments, respectively, of PTEC. The Operetta high content imager and Columbus image analysis server (both PerkinElmer, Waltham, MA, US) were used for imaging and analyzing.

### 2.6 Paraffin embedding and sectioning

Matrigel‐coated beads covered with cells were harvested, fixed, washed and encapsulated in 4% low melting agarose. Half an hour after gelation, agarose blocks containing beads were dehydrated and subsequently embedded into paraffin. The samples were sectioned at 4µm thickness using a microtome (Leica RM2255). After removing the paraffin by Xylene (Sigma‐Aldrich), the sections were heated in Target Retrieval solution (Dako) at 96°C in a water bath for 30 minutes for antigen retrieval and stained with antibodies.

### 2.7 Flow Cytometry

Adherent cells were dissociated to a single‐cell suspension using Trypsin/EDTA 0.5% (Thermo Fisher) and discriminated alive cells and dead cells using LIVE/DEAD™ Fixable Dead Cell Stain Kit (Thermo Fisher) for 30 min at 4°C. For labeling of intracellular antigen, cells were permeabilized using Phosflow Perm Buffer II (BD Biosciences) for 15 minutes at RT, incubated with primary antibodies for 30 minutes, and further for another 30 minutes with secondary antibodies. The dilution factor for primary antibodies was 1:100 or according to the manufacturer's instructions, and for secondary antibody it was 1:1000. Labeled cells were measured using MACSQuant Analyzer (Miltenyi Biotec), and data was analyzed with FlowJo software. All samples were performed in duplicates and all experiments were repeated 3 times. Antibodies used were SIX Homebox 2 (SIX2) (H00010736‐M01, Abnova), Receptor tyrosine kinase (RET) (53164, LSBio), Jagged 1 (JAG1) (ab7771, Abcam), Wilms’ tumor protein 1 (WT1) (sc192, SCBT), AQP1 (20333–1‐AP, Proteintech), Na⁺/K⁺‐ATPase (ab76020, Abcam), Megalin/LRP2 (ab76969, Abcam), SGLT2 (ab58298, Abcam), LTL (FL‐1321, Vector Laboratories), organic cation transporter 2 (OCT2) (ab242317, Abcam), organic anion transporter 1 (OAT1) (LS‐B10034, LSBio), organic anion transporter 3 (OAT3) (ab247055, Abcam), AF647 Anti P‐Glycoprotein (P‐gp) (ab253265, Abcam), multidrug resistance protein 2 (MRP2) (MA1‐26535, Thermo Fisher).

### 2.8 Transmission electron microscopy

Matrigel‐coated beads covered with cells were harvested, rinsed with PBS and fixed with 2.5% glutaraldehyde (Serva, Heidelberg, Germany) in 0.1 M sodium cacodylate buffer (Serva, Heidelberg, Germany) for 30 min at RT and stored at 4°C. The samples were postfixed with 1% osmium tetroxide (Electron Microscopy Sciences, Hatfield, USA) and 0.8% potassium ferrocyanide II (Roth, Karlsruhe, Germany) in 0.1 M cacodylate buffer for 1.5 h and embedded in agarose overnight. After cutting the agarose in smaller blocks, the samples were dehydrated in a graded ethanol series and transferred to Epon resin (Roth, Karlsruhe, Germany). Finally, ultrathin sections of the samples (70nm) were stained with uranyl acetate, and lead citrate. The examination was carried out with a Zeiss EM 906 electron microscope at 80kV acceleration voltage (Carl Zeiss, Oberkochen, Germany).

### 2.9 Glucose assay

Glucose uptake of cells on Matrigel‐coated beads was measured through cellular uptake of 2‐NBDG (2‐(N‐(7‐Nitrobenz‐2‐oxa‐1,3‐diazol‐4‐yl) Amino)‐2‐Deoxyglucose) (Thermo Fisher). Matrigel‐coated beads for assay were washed twice with DMEM without glucose, incubated with 400µM 2‐NBDG for 30 minutes in presence or absence of 1µM Dapagliflozin inhibitor. Beads were washed twice with DMEM without glucose before cells were harvested from beads using Trypsin/EDTA. Uptake of 2‐NBDG was measured using MACSQuant Analyzer (Miltenyi Biotec) and data analyzed with FlowJo software.

### 2.10 Albumin assay

Cellular endocytosis of albumin was investigated through application of different concentrations of FITC‐Albumin (Abcam) to cells on Matrigel‐coated beads. Matrigel‐coated beads for the assay were incubated with serum‐free medium with/without 50µg/ml, 100µg/ml, 500µg/ml, 1mg/ml FITC‐Albumin for 2 hours at 37°C. For immunofluorescence staining, cells were fixed using BD Cytofix, embedded into paraffin, sectioned and analyzed by Operetta high content imager and Columbus image analysis server (both PerkinElmer, Waltham, MA, US). For FACS analysis, cells were harvested from Matrigel‐coated beads using Trypsin/EDTA and albumin uptake was assessed using MACSQuant Analyzer and data analyzed with FlowJo software.

### 2.11 Organic anion uptake assay

To investigate organic anion transport by the basolateral organic anion transporters OAT1 and OAT3, an assay was performed using the fluorescent anion 6‐Carboxyfluorescein (6‐CF) (Thermo Fisher), a tracer dye, as described by Lawrence et al..^13^ Briefly, d16 cells on Matrigel‐coated beads were incubated with 50µM 6‐CF for 40 minutes in presence or absence of 2.5mM Probenecid inhibitor (Sigma‐Aldrich). Beads were washed twice with PBS before cells were harvested from beads using Trypsin/EDTA. Uptake of 6‐CF was measured using MACSQuant Analyzer (Miltenyi Biotec) and data analyzed with FlowJo software.

### 2.12 Organic cation uptake assays

Organic cation uptake by PTEC was investigated using the fluorescent cationic molecule DAPI, transported into live PTEC through the organic cation transporter 2 (OCT2) as described by Lawrence et al..^13^ Shortly, uptake of 1µM DAPI for 90 minutes by OCT2 was evaluated in presence or absence of Metformin and Cimetidine inhibitors (Abcam). Additionally, the activity of renal OCT2 influx protein was checked through the exposure to 5µM fluorescent OCT substrate 4‐Di‐2‐ASP (Sigma‐Aldrich) for 15 minutes in presence or absence of 15mM OCT inhibitor Tetrapentylammonium chloride (TPA) (Santa Cruz) as demonstrated by Jansen et al..^14^ The uptake of these fluorescent substrates was measured using MACSQuant Analyzer (Miltenyi Biotec) and data analyzed with FlowJo software.

### 2.13 Transport assay of renal efflux P‐Glycoprotein

ABC transporter permeability (P)‐glycoprotein (*ABCB1*; MDR1/P‐gp) of d16 cells on Matrigel‐coated beads was evaluated using Calcein accumulation.^13^ Thus, cells were incubated with 100nM Calcein, AM, cell‐permeable dye (Thermo Fisher) for 15 minutes in presence or absence of 40µM P‐gp inhibitor PSC‐833 (MedChemExpress). Calcein retention was measured using MACSQuant Analyzer (Miltenyi Biotec) and data analyzed with FlowJo software.

### 2.14 Cytotoxicity assay

PTEC were harvested from the beads at d14 using TrypLE^TM^ Express (Gibco) and reseeded at a concentration of 50.000 cells/well into Geltrex‐coated 96 wells. After incubation at 37°C for 2 days, PTEC medium was replaced. Confluent monolayers of cells were treated with various concentrations of Cisplatin (Sigma) from 50µM to 400µM for 6 hours in triplicates. Nephrotoxicity of Cisplatin was measured by MTT assay (Sigma‐Aldrich). After 6 hours of Cisplatin treatment, medium was changed to fresh medium, supplemented with 0.5mg/ml MTT labeling reagent yellow tetrazole and incubated in the dark for further 4 hours. NAD(P)H‐dependent cellular oxidoreductase enzymes of living cells were capable of reducing tetrazolium dye MTT to its purple insoluble formazan. Insoluble formazan was dissolved by incubating cells with solubilization solution overnight. The absorbance or optical density (OD) was measured at 570nm and 650nm using a microplate reader (Spectra max 384). Cisplatin untreated cells were used as negative controls.

Cell viability was calculated by formula:

$$\frac{OD\left(570\right)‐OD\left(650\right)of\;treated\;sample}{OD\left(570\right)‐OD\left(650\right)of\;untreated\;sample}\times 100$$

### 2.15 Transepithelial electrical resistance

Transepithelial electrical resistance (TEER) of matured PTEC monolayers on Transwell inserts (cellQART) was measured using an EVOM3 Ohmmeter. TEER was measured according to manufacturer´s instruction. D14 cells were harvested from Matrigel‐coated beads and reseeded on 24‐Transwell insert with a density of 50.000 cells per insert. 2 days after cell seeding, epithelial resistance was measured daily. To obtain TEER value (Ω.cm^2^), blank resistance (insert with media only) was subtracted from the measured resistance and afterward multiplied by the surface area.

### 2.16 Statistical analysis

Cell culture on Matrigel‐coated beads were performed in duplicates and further cell characterization and functionality tests were repeated 3 times (*n* = 3). Statistical analysis was performed by GraphPad Prism 5 (GraphPad Software, La Jolla, US). Statistical significance was calculated by two‐way ANOVA.

## 3 RESULTS

### 3.1 Matrix‐coated alginate beads offer an adjustable surface for hPSC expansion and renal differentiation in a dynamic culture system

The differentiation protocol (Figure 1(A)) was applied to three different hPSC‐lines, the fibroblast derived hiPSC‐line WISCi004‐A, the urinary cell derived hiPSC‐line BCRTi005‐A, and the human embryonic stem cell (ESC)‐line WAe001‐A. Cells attached to >90% of the beads with optimized inoculation density and culture medium volume (Figure 1(B)). 4 days after seeding hPSC, fold expansion was 6.2 (BCRTi005‐A), 6.7 (WISCi004‐A), and 9.7 (WAe001‐A), respectively (Table 1, Figure 1(C)).

**TABLE 1:** Cell yields during hPSC differentiation on Matrigel‐coated beads in the bioreactor

	Day ‐4	Day 0	Day 4	Day 8	Day 20
	hPSC	hPSC	metanephric mesenchyme	renal vesicle progenitors	PTEC
Cell lines	cells/cm^2^	cells/cm^2^	cells/cm^2^	cells/cm^2^	cells/cm^2^
BCRTi005‐A	6.5 × 10^4^	4 × 10^5^	4.1 × 10^5^	4.3 × 10^5^	6 × 10^5^
WISCi004‐A	6.5 × 10^4^	4.4 × 10^5^	4.3 × 10^5^	3.8 × 10^5^	6 × 10^5^
WAe001‐A	6.5 × 10^4^	6.3 × 10^5^	4.9 × 10^5^	5.4 × 10^5^	6.6 × 10^5^

Attachment and expansion of hPSC in 4 days was immediately followed by successive differentiation into mesoderm, renal progenitors and PTEC (Figure 1(A)).^6^ After an initial increase in cell numbers during expansion of hPSC (d0), a steady number of renal progenitor cells was maintained until d8 (Table 1, Figure 1(C)). After subsequent differentiation and expansion of PTEC (Figure 1(A)), the cell numbers further increased until d20 (Table 1). More specific, when a starter culture of 2.6 × 10^6^ hPSCs were cultured on 40cm^2^ of beads, around 24‐26 × 10^6^ PTEC were obtained on d20. In comparison, in parallel static culture on Matrigel‐coated 6‐well plates, 4 to 5 times fewer renal progenitor cells were obtained at d8 (<1 × 10^5^ cells/cm^2^), and at d20 (<1.4 × 10^5^ cells/cm^2^) for all cell lines, when the starting cell numbers were same as in the fluidic culture. Toluidine blue stained histological section of a Matrigel‐coated alginate bead at d16 showed a PTEC monolayer on the bead surface (Figure 1(D)).

In summary, starting with hPSC, the average number of cells increased reproducibly 9.2 and 10.2 times using hiPSC lines and the ESC line, respectively, after expansion and differentiation into PTEC (Figure 1(C)).

### 3.2 Differentiation of hPSC to PTEC on matrix‐coated alginate beads recapitulates the developmental stages of nephrogenesis

**FIGURE 2:**
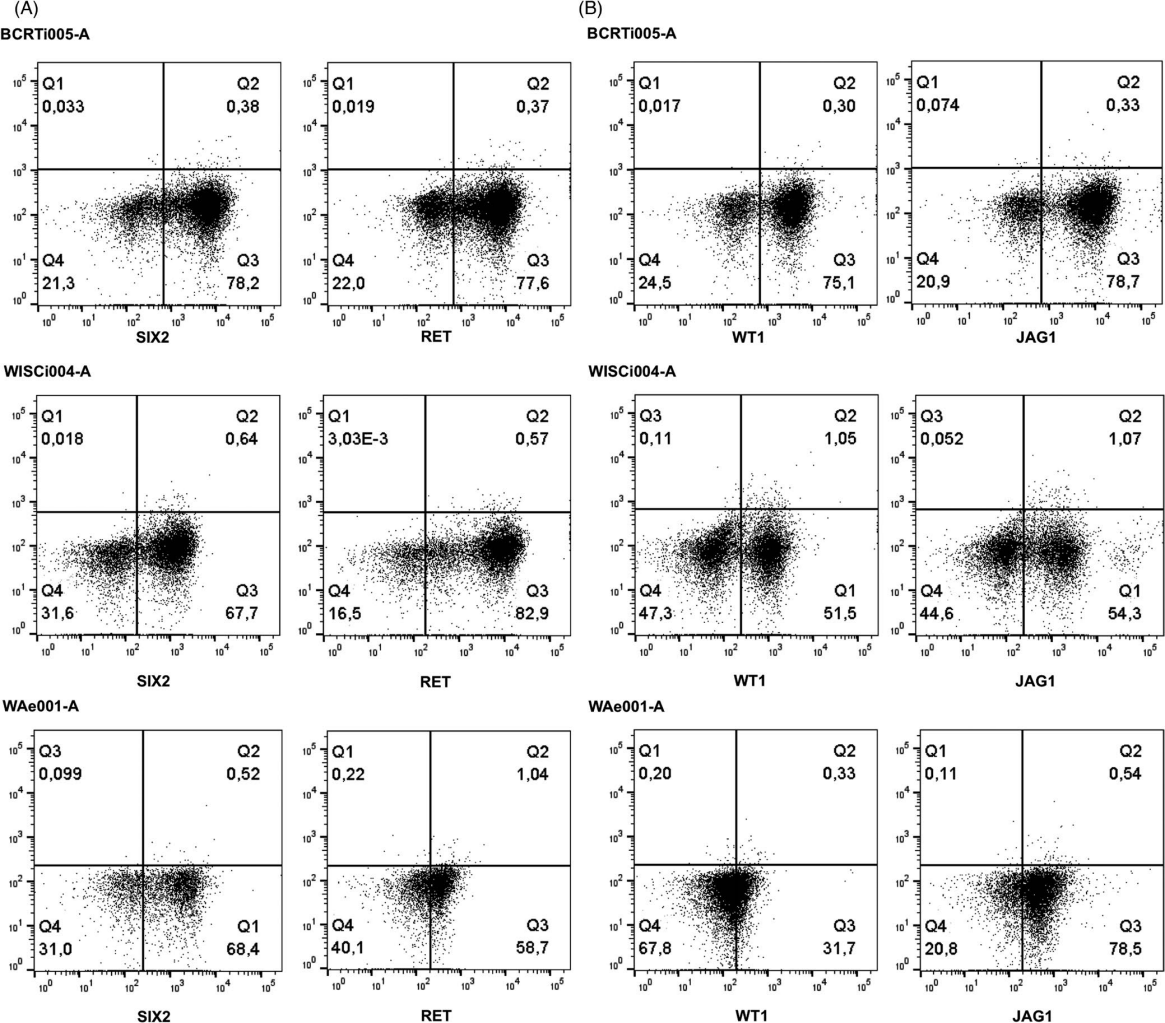
Efficiency of renal progenitor cell induction. Flow cytometry analysis of d4 and d8 cells generated from hPSC lines BCRTi005‐A, WISCi004‐A and WAe001‐A showed a high percentage of (A) SIX2 and RET positive cells on d4; (B) WT1 and JAG1 positive cells on d8

hPSC treatment with Activin A, Retinoic Acid and BMP4 increased SIX2 expression, a key transcription factor for metanephric mesenchyme.^14^ About 80% of BCRTi005‐A‐derived cells and 68% of WISCi004‐A and WAe001‐A cells showed SIX2 expression on d4 (Figure 2(A)). These percentages decreased with further specification in the renal lineage, to 50% on d8 for all three pluripotent cell lines. Around 60% of the cells expressed RET, an indicator of ureteric bud formation,^15^ in all three cell lines on d4 (Figure 2(A)). Occurrence of a RET positive population already on d4, before inducing ureteric bud by GDNF, could be explained by SIX2 + cells producing GDNF locally for ureteric bud formation and growth.^14^ The nephron progenitors emerging by d4 progressed to the renal vesicle stage as visualized by the appearance of WT1 and JAG1 by d8 (Figure 2(B)). The three hPSC lines showed a higher population of JAG1 than WT1. The Notch ligand JAG1 is typically expressed in renal vesicle cells closest to the ureteric bud tip as well as in the prospective proximal tubular cells.^16^ Its consistent appearance at d8 indicates initiation of PTEC differentiation. The successive appearance of metanephric mesenchyme, ureteric bud and renal vesicle cells during the induced differentiation process is consistent with renal development and the results obtained in static culture in previous work^6^ (Figure S1).

### 3.3 hPSC‐derived renal progenitors give rise to polarized PTEC layers on matrix‐coated alginate beads

**FIGURE 3:**
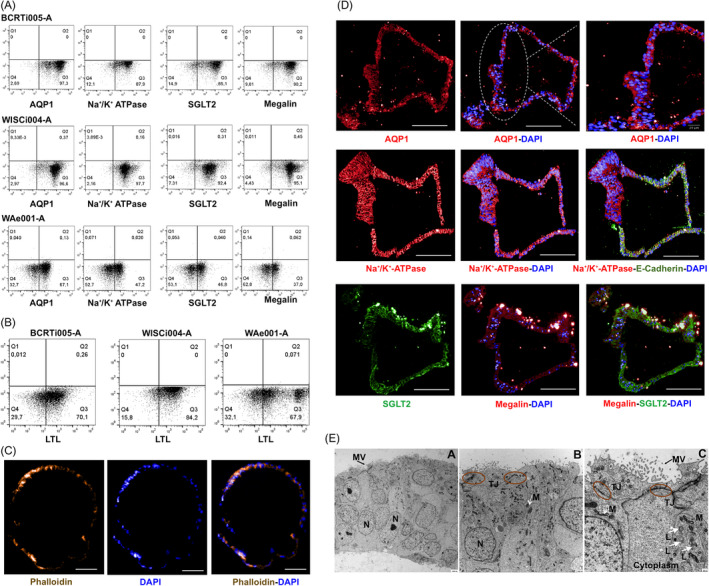
Assessment of hPSC‐derived PTEC. hPSC were seeded, expanded and differentiated in suspension on Matrigel‐coated alginate beads for scalable automated manufacture of PTEC (A) Flow cytometry analysis of cells on d16 post‐differentiation induction for AQP1, Na⁺/K⁺‐ATPase, SGLT2, Megalin/LRP2. Starting cells were the hiPSC lines BCRTi005‐A, WISCi004‐A and the ESC‐line WAe001‐A, respectively; (B) Flow cytometry analysis of d16 cells showing LTL and (C) Fluorescence microscopy for Phalloidin (yellow); DAPI (blue) shows nuclei. Scale bar = 50µm; (D) Immunofluorescence analysis of d20 cells showed expression of AQP1, Na⁺/K⁺‐ATPase, Megalin/LRP2 (all in red); SGLT2, E‐Cadherin (CDH1) (green). Scale bar = 100µm; (E) Transmission electron microscope images of d16 cells showed apical‐basal polarization (A). Basal side oriented toward Matrigel beads. Microvilli on apical side (MV). (A‐C) Tight junction (TJ); nuclei (N), mitochondria (M) and lysosomes (L)

**FIGURE 4:**
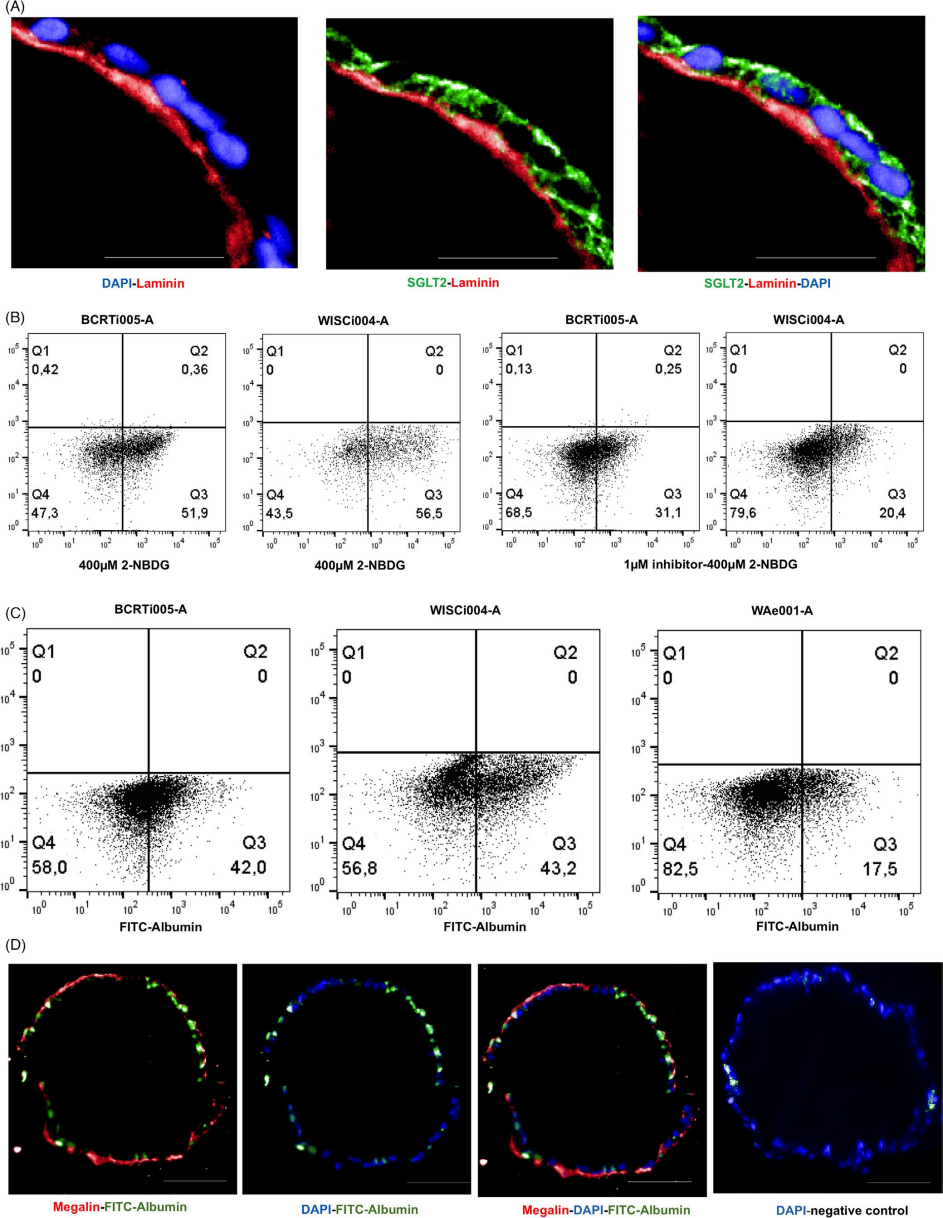
Functional analysis of hPSC‐derived PTEC (a‐b) SGLT2 localization and Glucose uptake of d16 PTEC. (A) Immunofluorescence staining for SGLT2 (green), Laminin (red); Scale bar = 20µm; (B) Flow cytometry analysis of 400µM 2‐NBDG uptake in absence or presence of 1µM Dapagliflozin of d16 PTEC; (C) Flow cytometry analysis for 1mg/ml FITC‐Albumin‐endocytosis of d16 PTEC; (D) Immunofluorescence staining for Megalin/LRP2 and FITC‐Albumin of d16 PTEC; Scale bar = 50µm

PTEC medium was used to further differentiate renal progenitors starting on d8 into PTEC. Differentiation efficiency was assessed on d10, d12, d14, d16 and d20. Cells started to express PTEC markers like AQP1, Megalin/LRP2, and SGLT2 as early as d12. On d16, above 80% of cells derived from the hiPSC and around 50% of the ESC‐derived cells expressed PTEC‐specific markers AQP1, Na⁺/K⁺‐ATPase, as well as protein transporters including Megalin/LRP2 and SGLT2 (Figure 3(A)). Around 40–60% d16 cells also expressed other transporter proteins required for transepithelial movement of organic ions, such as OCT2, OAT1, OAT3 on the basolateral side; P‐gp and MRP2 on the apical side (Figure S2). For hiPSC‐derived PTEC, expression of these markers was stable until d20 while the ESC‐derived PTEC showed decreasing expression over time. Lotus lectin (LTL) that binds to Fucose on microvilli of PTEC and is an indicator for mature PTEC^17^ was also abundant (around 70%) on d16 (Figure 3(B)). Moreover, Phalloidin allowed visualization of actin bundles, suggesting the presence of microvilli^18^ on the hPSC‐derived PTEC, confirmed by immunofluorescence analysis on d16 (Figure 3(C)). In contrast, in static culture with the same medium and the same protocol, around 60% of the cells expressed AQP1 and 55% were positive for LTL on d16 (Figure S3(A)). When cultivated on Laminin 521 during static culture, PTEC expressed LTL (66%) and AQP1 (82%) ((Figure S3(B)), which was localized on the membrane but not homogenously in the culture; however, they showed a cuboidal morphology (Figure S3(C)) typical of tubular epithelial cells in the proximal tubule. The expression data obtained by flow cytometry were confirmed by immunofluorescence microscopy of cells on Matrigel beads (Figure 3(D), Figure S4). In addition, the basement membrane was explored by analyzing Laminin localization in combination with the typical apical membrane localized cotransporter SGLT2. Both proteins showed the expected apico‐basal localization in hPSC‐derived PTEC (Figure 4(A)).

To check further polarity and ultra‐structures of PTEC, cells were visualized on d16 by transmission electron microscopy (TEM) (Figure 3(E)). Polarization was confirmed with the basement side resting on beads and the apical side with numerous microvilli. Large numbers of mitochondria were visible, a physiologically relevant feature of kidney proximal tubules. PTEC also contained many large lysosomes of different stages. Proximal tubular lysosomes are responsible for catabolizing proteins such as albumin after uptake from the glomerular filtrate and are present at high numbers in the cytoplasm.^19^ Tight junctions were well‐developed between PTEC.

### 3.4 hPSC‐derived PTEC are capable of active reabsorption

Selective uptake of glucose and albumin from glomerular filtrate is a main function of PTEC in the kidney. The uptake capacity of substances was investigated on d16 post‐differentiation induction. In PTEC, SGLT2 is responsible for 90% of the glucose‐reabsorption from the glomerular filtrate *in vivo*. This transporter is expressed on the apical side of PTEC (Figure 4(A)). d16 cells were incubated with 400µM 2‐Deoxy‐2‐[(7‐nitro‐2,1,3‐benzoxadiazol‐4‐yl)‐amino]‐D‐glucose (2‐NBDG) for 30 minutes in presence or absence of 1µM Dapagliflozin, a selective inhibitor of SGLT2. 2‐NBDG was taken up by 50% of cells (Figure 4(B)). In the presence of Dapagliflozin, 2‐NBDG uptake decreased by 50% (Figure 4(B)).

Receptor‐mediated albumin endocytosis by PTEC is carried out by Megalin/LRP2. Albumin uptake of d16 cells were maximum, by 20% ‐ 40% of PTEC when cells were incubated with 1mg/ml FITC‐Albumin for 2 hours (Figure 4(C)). Fluorescence microscopy analysis showed expression of Megalin/LRP2 and endocytosis of FITC‐Albumin (Figure 4(D)).

The ability of d16 cells on Matrigel‐coated beads to take up organic cations was investigated using the fluorescent cationic molecule DAPI, which can be transported into live PTEC through the basolateral organic cation transporter 2 (OCT2).^13^ Around 45% of d16 cells were able to uptake the fluorescent OCT substrate 4‐Di‐2‐ASP and about 26% showed DAPI transport. In the presence of OCT2 inhibitors, uptake of both substrates decreased appreciably (Figure S5(A,B)). The fluorescent anion 6‐Carboxyfluorescein (6‐CF), a tracer dye, was used to investigate organic anion transport by the basolateral organic anion transporters OAT1 and OAT3.^13^ Activity of OAT1/OAT3 was determined as probenecid‐sensitive fluorescein uptake since only around 8% of the inhibitor‐treated cells took up 6‐CF as opposed to untreated cells, where up to 50% cells were capable of 6‐CF uptake (Figure S5(C)). PTEC were incubated with Calcein AM and an increase in fluorescence intensity was seen due to the cell‐permeant nature of the dye. To investigate if the efflux of the dye was mediated by P‐gp, cells were treated with the inhibitor PSC‐833. The fluorescence intensity of Calcein AM was unchanged indicating that the P‐gp (Figure S5(D)), though expressed on d16 cells, was not fully functional.

### 3.5 Modeling drug‐induced nephrotoxicity in hPSC‐derived PTEC

**FIGURE 5:**
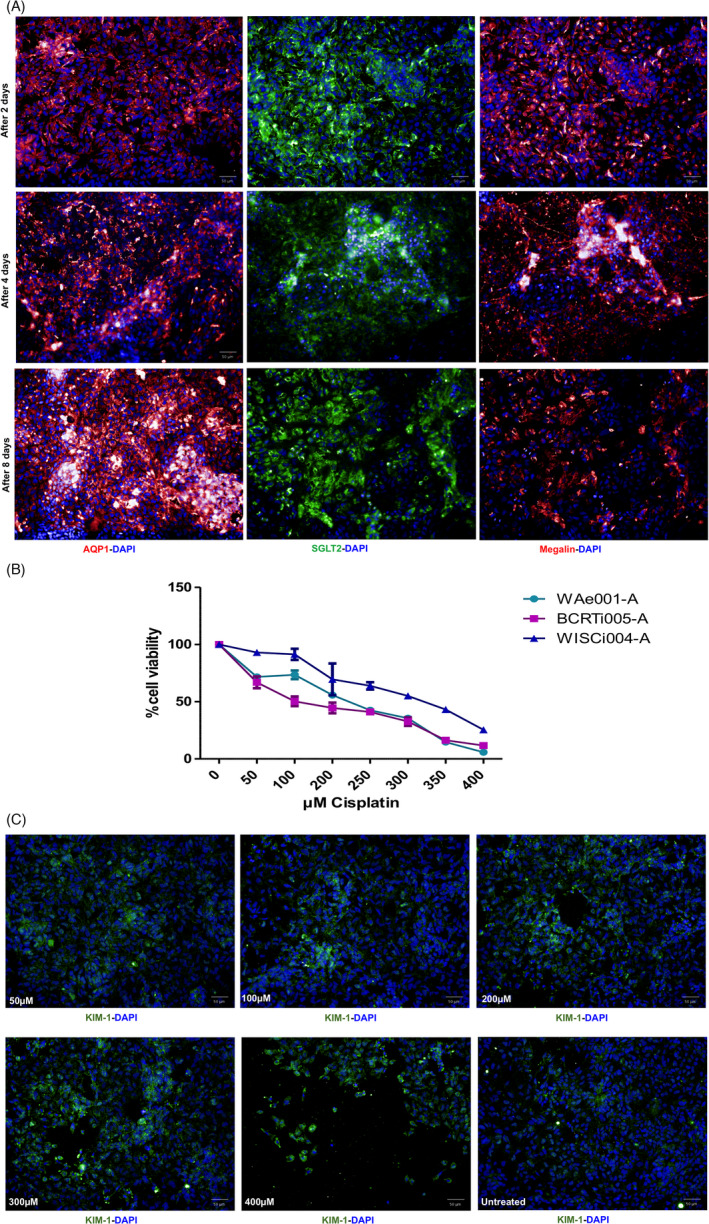
Cisplatin‐induced cytotoxicity on harvested hPSC‐derived PTEC. (A) Immunofluorescence showed expression of AQP1, Megalin/LRP2 (both in red), and SGLT2 (green) in PTEC harvested from Matrigel beads on d14 and cultured on 96‐well plates after 2 days, 4 days and 8 days in 2D culture. Scale bar = 50µm; (B) MTT assay showed concentration – dependent effects on cell viability after 6 hours of treatment of PTEC derived from WAe001‐A, BCRTi005‐A, and WISCi004‐A; (C) Immunofluorescence showed expression of KIM‐1 on BCRTi005‐A‐derived PTEC treated with Cisplatin for 6 hours. Scale bar = 50µm

PTEC were harvested from Matrigel‐coated beads on d14, post‐differentiation and reseeded on Geltrex‐coated 96‐well plates. The cells maintained expression of AQP1, SGLT2, Megalin/LRP2 on d2, 4 and 8 post‐seeding (Figure 5(A)) and formed a tight epithelial monolayer on transwell inserts within the first 2 days of seeding (Figure S6). At d22, a week of culture on the transwell insert later, the transepithelial resistance of PTEC stabilized at 90 Ωcm2, which is very similar to the TEER values historically demonstrated by Whitin et al. for HK‐2 cells (100Ωcm2).^20^ 2 days after reseeding on Geltrex‐coated 96‐well plates, cells were treated with Cisplatin for 6 hours showing a concentration dependent reduction in viability (Figure 5(B)). For all three cell lines used, PTEC started showing cytotoxic effect of Cisplatin at 50µM and a complete deterioration in cell viability at 400µM (Figure 5(C)). Sensitivity with Cisplatin of PTEC induced from WAe001‐A and BCRTi005‐A was quite similar and higher than of PTEC induced from WISCi004‐A. 50% reduction of cell viability in WISCi004‐A, WAe001‐A, and BCRTi005‐A‐derived PTEC was at 300µM, 200 µM, and 100 µM, respectively. KIM‐1 was detected by immunofluorescence staining on samples treated with Cisplatin for 6 hours (Figure 5(C)).

## 4 DISCUSSION

To establish an effective, economical, simple and reproducible protocol for expansion and differentiation of hPSC‐derived PTEC, a platform was developed using a levitating fluidic bioreactor together with an adjustable surface for cell adhesion, proliferation and directed differentiation based on alginate beads coated with Matrigel. The system allows expansion and differentiation without further cell processing in a single culture system. Cell yield and differentiation efficacy were compared with conventional cultivation in static culture on Matrigel‐coated polystyrene.^6^ Both, expansion rates and yield of PTEC was 2 times higher for hiPSC lines and 2,2 times for WAe001‐A hPSC‐lines, when expanded on alginate beads in the CERO bioreactor compared to static culture in the same medium and timeline. Although reproducible for the three selected hPSC‐lines, there was variability detectable between hiPSC lines and hESC line in terms of expansion and differentiation efficacy. This variability certainly did not affect functional differentiation, but it indicates the need for cell‐line specific optimization of cultivation conditions.

Biolevitation has been used previously to expand hPSC on Matrigel‐coated alginate beads.^21^ However, this method has never been utilized to obtain renal cells. Renal cells were previously differentiated and expanded in rotating‐wall vessels,^22^ in wave bioreactors^23^ or in specialized biorectors.^24^ An advantage of biolevitation and cell cultivation on floating beads is the continuous and adjustable flow exposure of the surface‐covering cells. Additionally it has been shown in many systems that flow promotes functional differentiation, *in vivo* cell behavior as well as it mimics the cellular environment by providing mechanical stimulation.^9^, ^25^, ^26^, ^27^, ^28^, ^29^ Thus, we believe that biolevitation in combination with alginate beads benefit differentiation of pluripotent stem cells into PTEC regarding both quality and quantity.

Despite the wide variety of applications of alginate hydrogel in cell encapsulation, cell transplantation, and tissue engineering, efficacy of using alginate hydrogel as three dimensional cell culture substrates have been only investigated for a few cell types such as mouse skeletal myoblasts,^30^ human and rat bone marrow stromal fibroblastic cells^31^ and hPSC.^9^ The high porosity of the biopolymer network in alginate hydrogels helps to establish a continuous exchange of nutrients, gasses, waste products and signaling molecules with cells grown on the hydrogel. Indeed, our results show that the formed PTEC monolayers polarize on the alginate bead with the vascular side forming a basal membrane and their apical microvilli exposed to the fluidic side. The functionality of such a polarized epithelium has also been demonstrated by the uptake of substrates by solute transporters, indicating a high‐level suitability for nephrotoxicity applications. Existing proximal tubule models rely on immortalized human proximal tubule cell lines like HK‐2, RPTEC‐hTERT and ciPTEC‐hTERT. The HK‐2 cells do not show the presence of organic ion transporters, whereas RPTEC and ciPTEC lines express them but require an additional lentiviral transduction of each transporter to be utilized for drug toxicity testing.^32^

Alginate can be easily modified in terms of stiffness and its surface chemically modified, to allow protein coating.^33^, ^34^ The utility of extracellular matrix (ECM)‐functionalized alginate hydrogel beads could be modified by selecting purpose‐specific matrices. ECM compositions including type IV collagen, entactin, heparan sulfate proteoglycan, and laminin differentially decorate the basement membrane of all segments along the nephron. For instance, laminin concentration in proximal tubules is 50% higher compared to the distal tubule.^35^ We chose Matrigel as a protein surface on alginate beads as this growth factor‐reduced ECM contains abundant animal glycoproteins (laminin, type IV collagen, heparan) and factors that mimic the basement membrane and help to support epithelial growth.^36^ For animal‐free cultivation, laminin or other matrix proteins may be used. Indeed, on 2D culture, when we used LN521 instead of Matrigel, differentiation efficacy increased slightly from 70% to 80% AQP1, 55% to 66% LTL positive cells by forming a typical cuboidal epithelial layer of PTEC on d16 (Figure S2(B), Figure S2(C)). Further optimization of alginate surface coating, for example with human kidney derived‐ECM or ECM‐components specific to the target nephron structure may enhance speed and efficacy of renal cell differentiation and maturation.^37^

Differentiation media to obtain PTEC from hPSC offer additional possibilities to modify and optimize the environment of the cells. We developed a simplified culture medium based on a medium used for growing rat PTEC on chitosan^38^ with significant variations. Specifically, we verified a basal medium with defined low glucose in normoglycemia ranges^39^, ^40^ and eliminated the xeno‐component, bovine pituitary extract and cholera toxin. In addition, hydrocortisone that sensitizes cells to EGF and increases proliferation was reduced to 1µM as PTEC are usually low‐proliferating cells *in vivo*. Thus, we designed a medium that is serum‐free and xeno‐free with minimal factors, offering a high controllability of the PTEC medium. Although the commercially produced REGM was used in previous studies to successfully generate renal vesicles and PTEC from hPSC,^6^ its disadvantage is the undisclosed growth factor concentration and the presence of serum with potential for batch variation and unsuitability for clinical application of the cell products. We observed continuous proliferation of PTEC after d16, when differentiation was completed. This is non‐physiological as epithelial cells of the proximal tubule under normal conditions do not proliferate. Not providing growth stimulation after a PTEC monolayer has formed may further improve physiological maturation.^41^

The successful large‐scale production of renal cells on matrix‐coated alginate beads by biolevitation offers an effective and low‐cost production of high quality PTEC derived from hPSC for multiple applications where there is a high demand, such as bioprinting, therapeutic application or as cellular components for tissue engineering. The monolayer coverage of the alginate beads with polarized PTEC and tight junctions furthermore may provide a system for high throughput screening of PTEC function where each bead mimics a tubular element with a basal tubular surface and an outer urinary surface. Additionally, even when harvested from the beads, these cells are capable of forming a tight epithelium within two days and express functional solute transporters, making them superior to existing immortalized cell lines.

In conclusion, we have successfully developed a platform technology for differentiation and expansion of hPSC‐derived PTEC in a serum‐free xeno‐free medium without the need for passaging, in a single cultivation unit, providing high cell numbers in a reproducible manner for multiple applications.

## 5 FUNDING STATEMENT

This work was supported by grants from the Federal Ministry of Education and Research (Bundesministerium für Bildung und Forschung ‐ BMBF): miro‐iPSC Profiler ‐ 01EK1612D, Bundesministerium für Wirtschaft und Technologie ‐ TIME ZF4274303, Charité 3R| Replace ‐ Reduce – Refine and the Vietnam International Education Development (VIED).

## AUTHOR CONTRIBUTION

*Thao Ngo* involved in methodology, investigation, formal analysis, visualization, writing original draft preparation. *Bella Rossbach* involved in methodology, investigation, formal analysis, visualization, validation, writing original draft preparation. *Isabelle Sébastien* involved in conceptualization, methodology, investigation. *Julia C*. *Neubauer* involved in conceptualization, methodology, resources. *Andreas Kurtz* involved in conceptualization, resources, supervision, funding acquisition. *Krithika Hariharan* involved in conceptualization, methodology, formal analysis, visualization, writing original draft preparation, supervision, project administration. All authors contributed to writing and review and editing of this manuscript.

## Supporting information

Fig S1

Fig S2

Fig S3

Fig S4

Fig S5

Fig S6

## Data Availability

The data that support the findings of this study, are available from the corresponding author upon reasonable request.
