# Supplementary figures and images for: Functional differentiation and scalable production of renal proximal tubular epithelial cells from human pluripotent stem cells in a dynamic culture system
Source: Cell Prolif. 2022 Jan 31;55(3):e13190. doi: 10.1111/cpr.13190 (PMC8891564; doi:10.1111/cpr.13190)

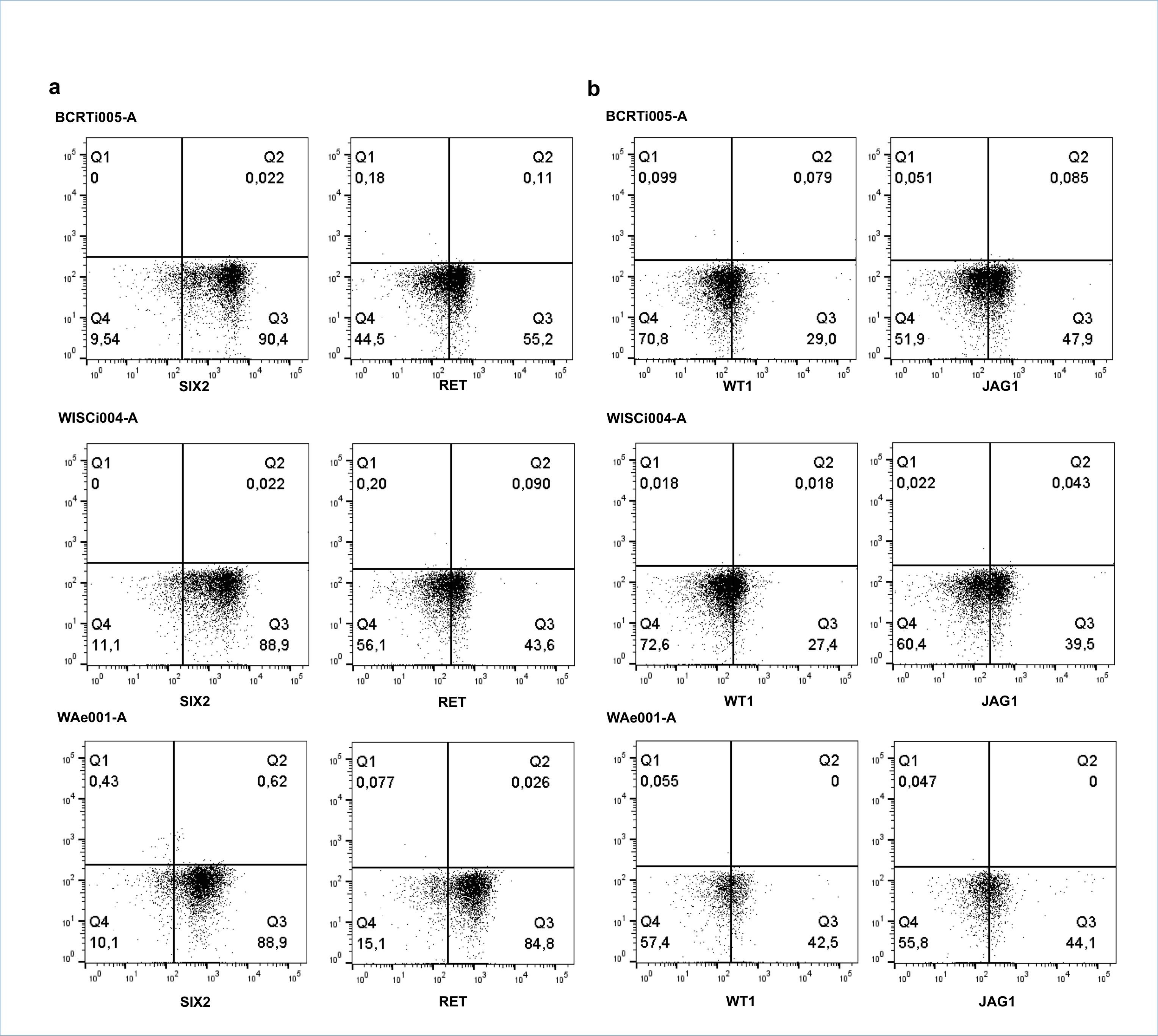

Supplement: Supplementary file 1 — Fig S1 [file CPR-55-e13190-s003.png]

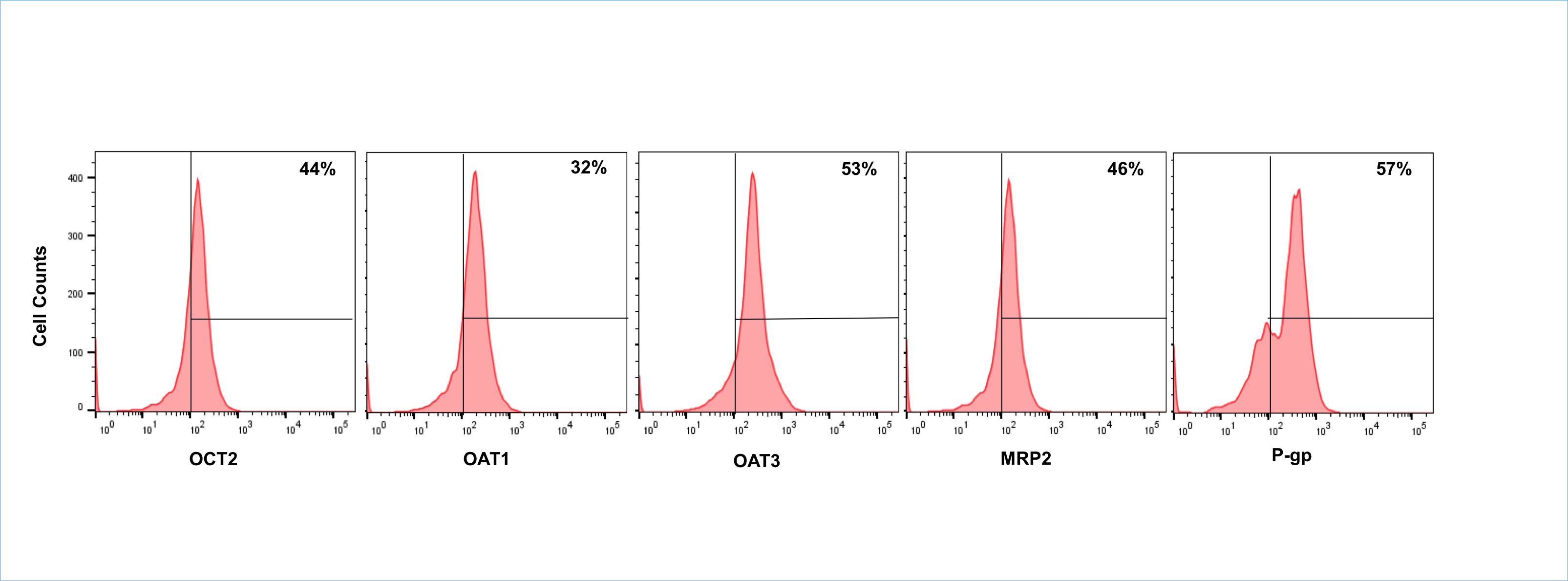

Supplement: Supplementary file 2 — Fig S2 [file CPR-55-e13190-s001.png]

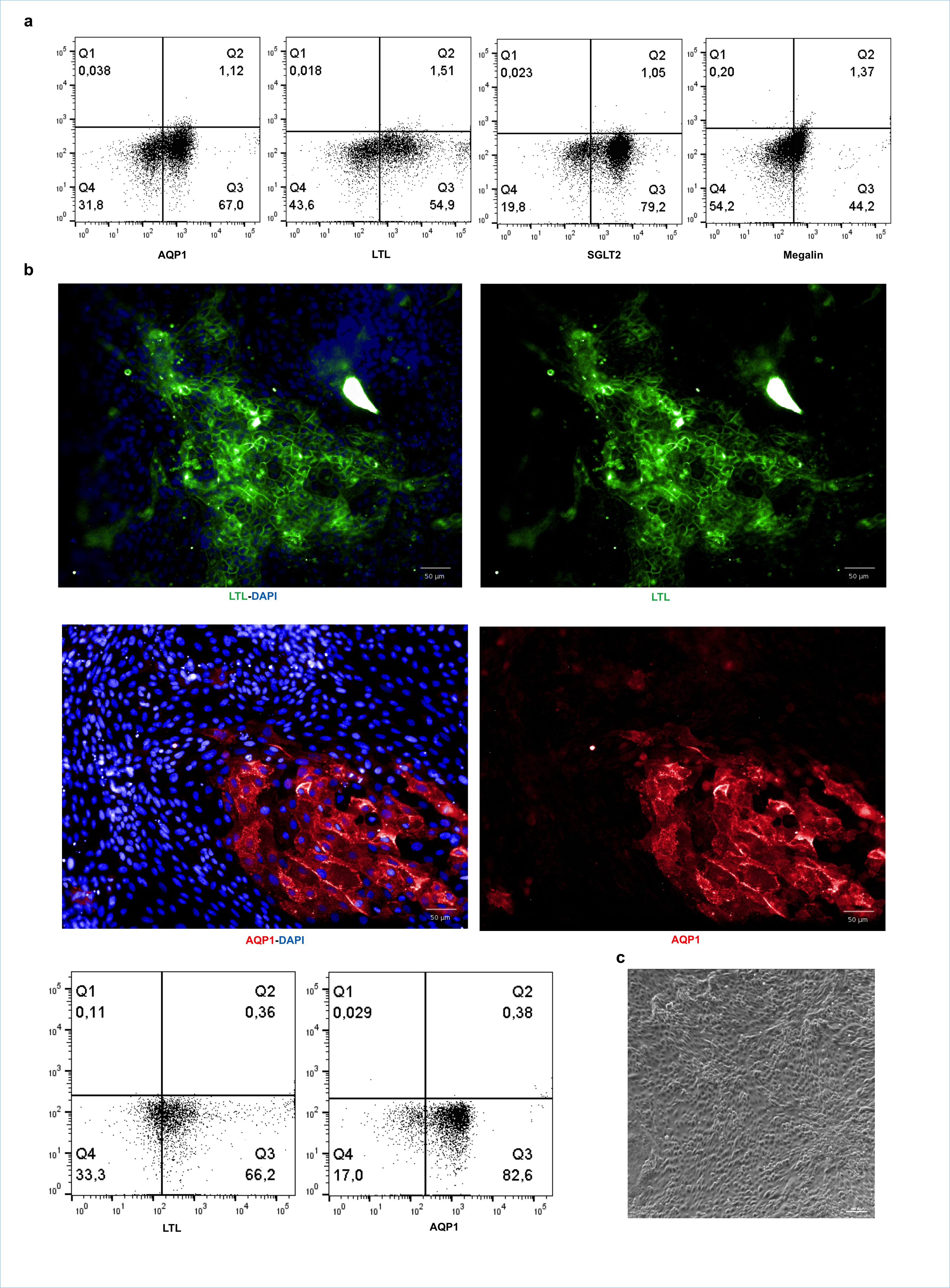

Supplement: Supplementary file 3 — Fig S3 [file CPR-55-e13190-s002.png]

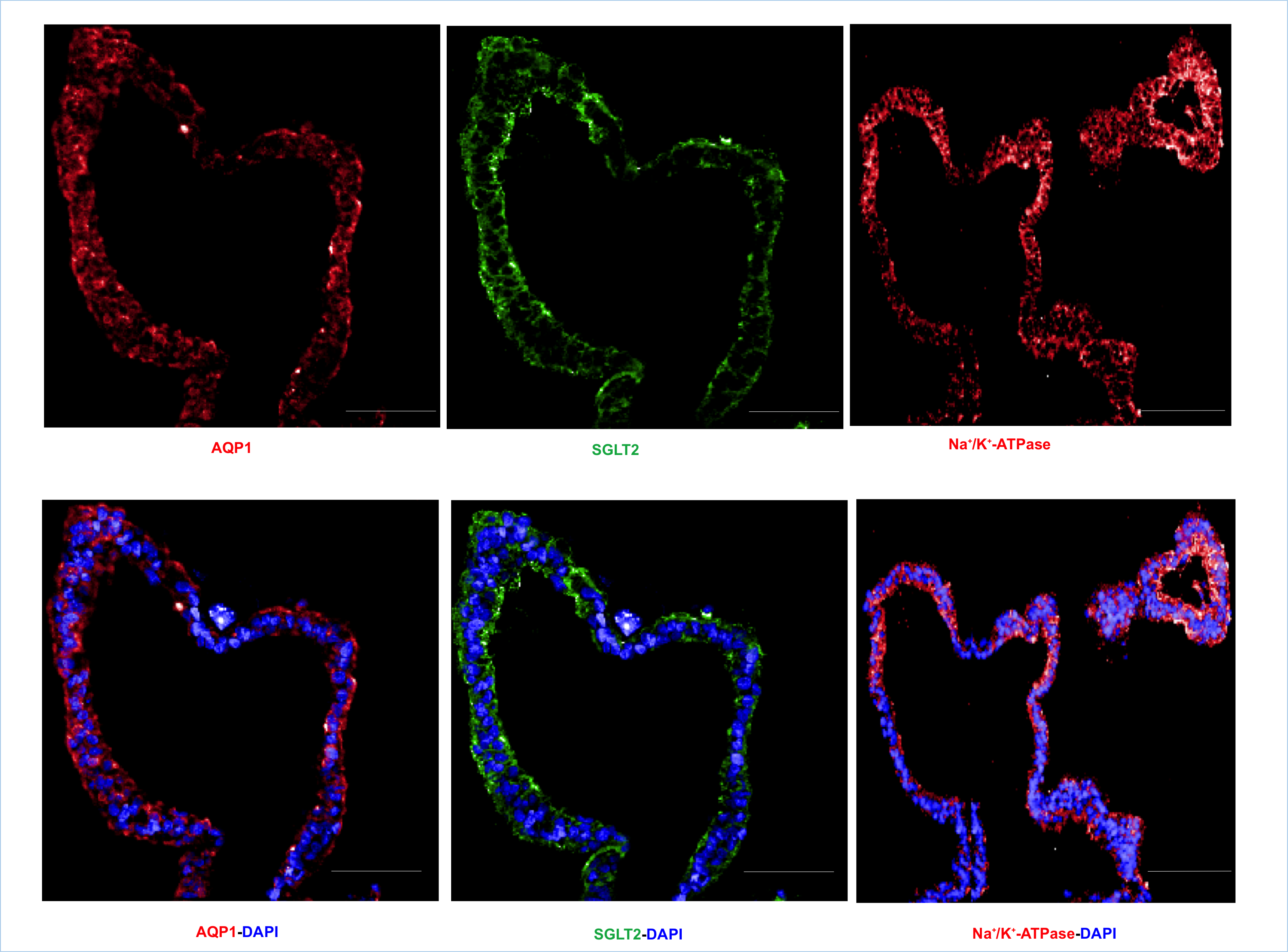

Supplement: Supplementary file 4 — Fig S4 [file CPR-55-e13190-s005.png]

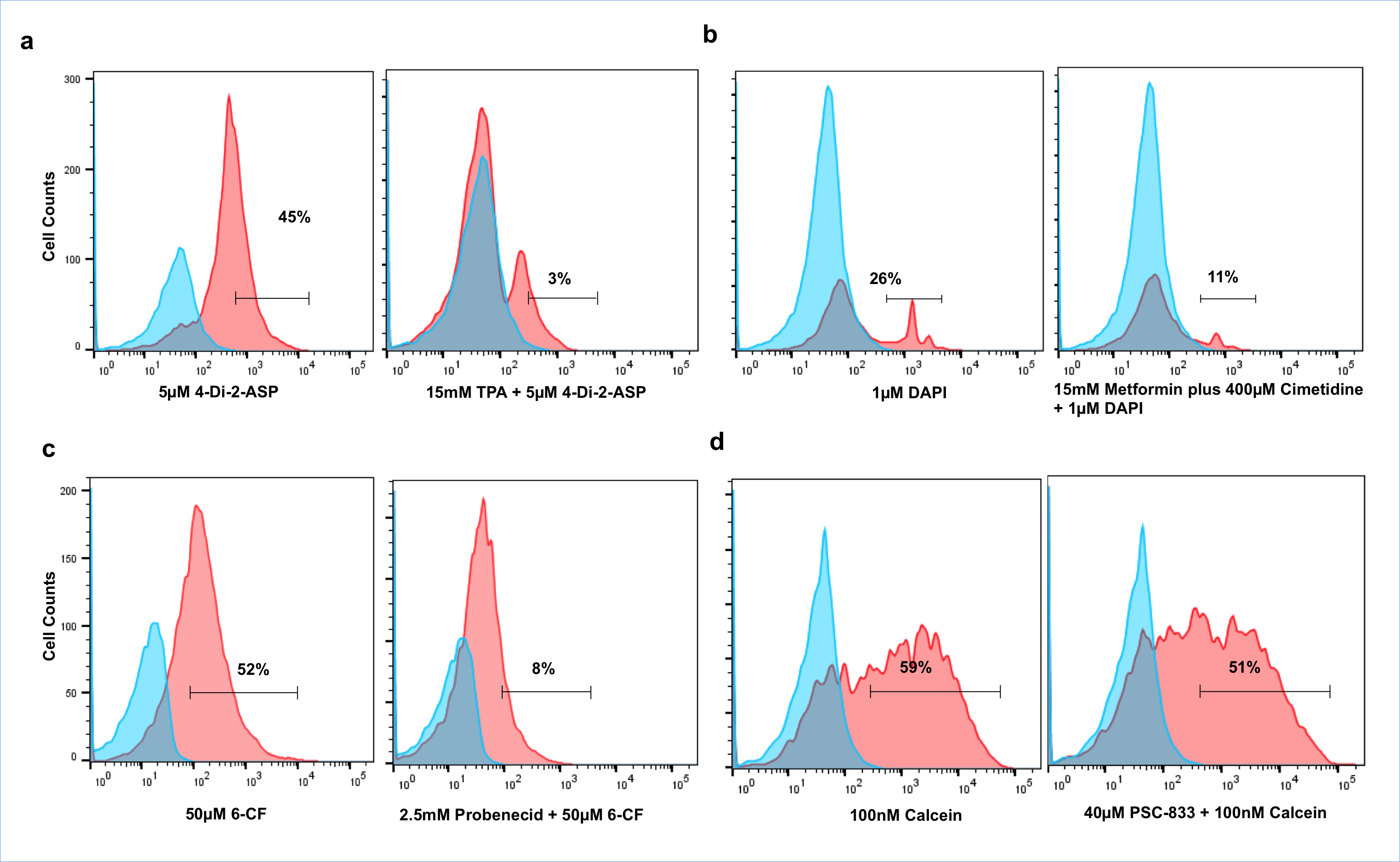

Supplement: Supplementary file 5 — Fig S5 [file CPR-55-e13190-s004.png]

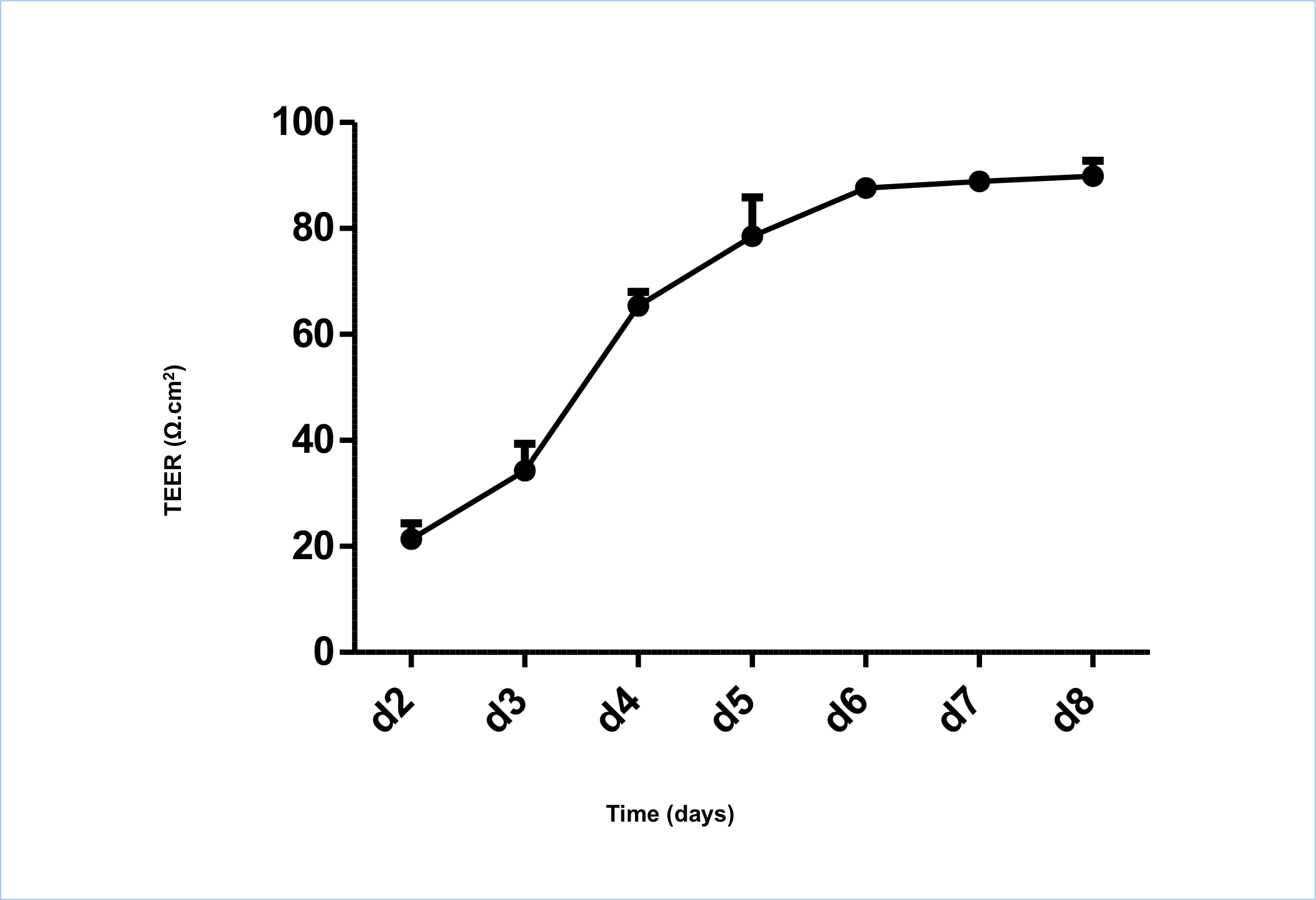

Supplement: Supplementary file 6 — Fig S6 [file CPR-55-e13190-s006.png]
